# The Cytotoxic Effect of Thermoplastic Denture Base Resins: A Systematic Review

**DOI:** 10.3390/jfb14080411

**Published:** 2023-08-03

**Authors:** Shankargouda Patil, Frank W. Licari, Shilpa Bhandi, Kamran H. Awan, Almir Badnjević, Valentina Belli, Gabriele Cervino, Giuseppe Minervini

**Affiliations:** 1 College of Dental Medicine, Roseman University of Health Sciences, South Jordan, UT 84095, USAsbhandi@roseman.edu (S.B.);; 2 Verlab Research Institute for Biomedical Engineering, Medical Devices and Artificial Intelligence, 71000 Sarajevo, Bosnia and Herzegovina; almir@verlabinstitute.com; 3 Department of Precision Medicine, Università degli Studi della Campania “Luigi Vanvitelli”, 80131 Naples, Italy; 4 Department of Biomedical and Dental Sciences, Morphological and Functional Images, University of Messina, G. Martino Polyclinic, 98100 Messina, Italy; 5 Multidisciplinary Department of Medical-Surgical and Dental Specialties, University of Campania, Luigi Vanvitelli, 80138 Naples, Italy; giuseppe.minervini@unicampania.it

**Keywords:** biocompatibility, cell viability, cytotoxicity, denture base, L929, polyamide, primary cell culture, thermoplastic denture base resins

## Abstract

Partial or complete dentures are constructed from thermoplastic resins that are thermally processed and molded. This review examines the presently available evidence for the cytotoxicity of thermoplasticized denture base resins on human gingival epithelial cells, adipose cells, and fibroblasts; human amnion fibroblasts; and mouse fibroblasts. Electronic searches were performed on PubMed, Scopus, Web of Science, and Google Scholar databases to identify relevant articles to be included in the review until September 2022. Clinical, in vivo, and in vitro studies in English language were searched for. The quality of the studies was assessed using the Toxicological data Reliability Assessment tool (ToxRTool) developed by the European Commission’s Joint Research Centre. GRADE assessment was used to evaluate the certainty of evidence. Seven in vitro studies were included in the review. The overall risk of bias was determined to be high, with the majority of studies assessed found to be reliable with restrictions or not reliable. Only two studies were considered reliable without restrictions based on ToxRTool assessment. The effect of thermoplastic denture base resins on viability and cell adherence of human gingival or amnion fibroblasts and mouse fibroblasts (L929s) is not significant. Conditioned media from unpolished specimens of resins were significantly more toxic to cultured cells than those from polished specimens. This may be of concern in cases of poor post-processing of dentures. Based on the limited evidence available, there is low-certainty evidence that thermoplastic denture base resins appear to be biocompatible and show insignificant cytotoxicity. Further well-designed trials adhering to standard reporting guidelines and using objective measures are necessary before outlining universal guidelines for best practice. Long-term in vivo and clinical assessment is necessary to corroborate laboratory findings with clinical outcomes. Denture base resins are in constant contact with oral tissues, and cytotoxic components released by the resins may irritate or inflame the tissues or provoke an allergic response.

## 1. Introduction

An aging population poses unique dental challenges. According to the World Health Organization, by 2030, one in six people will be over 60 years of age [1]. This shift in the distribution of population aging along with tooth loss will necessitate greater requirements and accessibility for dentures. In 2019, edentulism and tooth loss was ranked 22nd globally as the Level 4 cause of disability. They were also ranked 31st with a global prevalence of 352 million cases and 56th with a global incidence of 25 million cases [2]. Loss of tooth results in functional, aesthetic, as well as social impairments.

Replacement of missing teeth is mandatory to satisfy aesthetic and functional needs [3]. The intermediate medium between artificial teeth and the jaw is denture base material, which remains in direct contact with the oral tissues for a long time. The long-term incessant intraoral contact of denture prostheses necessitates an investigation into the biocompatibility of these materials. The oral environment and conditions such as pH, thermal changes, moisture, enzymes, and microflora can bring about changes in the chemical and physical properties of denture base materials [4].

Polymethyl methacrylate (PMMA) is the most commonly used material for the fabrication of dentures; though popular, it is far from an ideal denture base material. The most important disadvantage of a PMMA denture base is high fracture incidence [5].

The acrylic resin consists of powder and liquid components. The powder contains a colored polymer, and the liquid contains a clear, volatile, flammable monomer. This monomer is cytotoxic and perhaps genotoxic [6]. Heat-cured acrylic resins that are most commonly used for the construction of dentures release formaldehyde, methyl methacrylate, and benzoic acid, which are toxic and may elicit adverse reactions [7].

The monomer–polymer ratio and conversion highly influence the mechanical and biological properties of denture resin. Incomplete polymerization results in the presence of residual monomers in the denture base that may leach into saliva and other fluids [8,9]. These methacrylate monomers can cause irritation, inflammation of oral mucosa, or allergic reaction in patients and dental practitioners [10]. Allergic reactions are seen in around 0.7–2% of patients and dental staff [11]. To overcome the disadvantages of PMMA, various polymers have been developed such as polyamides, polycarbonate, polystyrene, nylon, epoxy resin, vinyl acrylic, and rubber graft copolymers.

Thermoplastic denture base resins were introduced to fabricate flexible dentures to avoid the unesthetic exposure of metal as in thermally polymerized conventional resin. They are used for fabricating temporary dentures and non-metallic clasp dentures (NMCDs) [12]. Though their elastic modulus is lower than conventional resins, their higher elasticity, fracture resistance, softness, and flexibility help in their application on abutments with a large undercut [13,14]. These resins are translucent and pink in color, thereby satisfying the natural aesthetic look by blending with the gingival tissue. These can be made thinner and lighter than conventional resins and have improved wearability. They have low allergenic risk, are resistant to acids and alkalis, and have a smoother surface [15]. Amongst various thermoplastic polymers, polyamide, polypropylene, and thermoplastic acrylic resins are commercially available for clinical use and fabrication of NMCDs.

Polyamide resins are polymerized by a condensation reaction between a diamine and a dibasic acid [16,17]. They have repeated units linked by amide bonds [18]. Applications of propylene include the fabrication of plastic, reusable equipment, and packaging due to its low melting point (~130 °C) and convenience of use [19]. Thermoplastic acrylic resins were developed to overcome the drawbacks of thermoplastic polymers such as difficulty in relining and repairing because of their weak bond with self-curing resin and teeth [20]. Polyamides fabricated by heat-injected molding technique have better dimensional stability and lower polymerization shrinkage of the resin [21]. These injection-molded polymers are free of monomers and are more flexible than PMMA; hence, they are used for the fabrication of flexible dentures and occlusal splints.

Various studies have evaluated physic mechanical properties such as flexural strength, impact strength, elastic modulus, hardness, color stability, and water sorption [12,22,23,24,25]. Dental materials are in constant contact and constantly interact with the oral tissues. Placed in a biological environment, a material is considered biocompatible when it does not produce adverse effects on contact with a living system [26]. Considering their several clinical applications, the biological and toxicological properties of thermoplastic resins are critical.

The oral cavity is a dynamic environment, subjecting dental materials to a variety of physical and chemical stresses. Denture base resins, in particular, come into direct contact with the oral mucosa, and any potential cytotoxic effects can have adverse implications for patient health and well-being. Assessing the cytotoxicity of these materials is crucial to ensure patient safety, prevent adverse reactions, and optimize the success of denture treatment outcomes. Cytotoxicity refers to the potential of a substance to cause damage or death to living cells. In the case of thermoplastic denture base resins, the release of potentially toxic substances from the material, such as residual monomers or other chemical components, may lead to cellular damage or inflammatory reactions within the oral tissues [23,27,28,29]. These cytotoxic effects can manifest as tissue irritation, allergic reactions, or even systemic effects if the released substances are absorbed into the bloodstream [30]. Considering the clinical implications of using thermoplastic denture base resins, it is paramount to conduct a systematic review to comprehensively evaluate the available evidence regarding their cytotoxic effects, consolidate the existing knowledge, and identify research gaps, which will help inform clinical decision-making.

To determine the biocompatibility of materials, in vitro cytotoxicity tests on cultured cells or tissue are essential to test the potential toxicity. Short-term and long-term cytotoxicity have been evaluated using direct or indirect contact tests using cultured human fibroblasts, epithelial cells, or mouse fibroblasts (L929s). This study aims to contribute to the understanding of the cytotoxicity associated with thermoplastic denture base resins, providing valuable insights for researchers, clinicians, and dental professionals. The findings of the systematic review can inform decision-making regarding the selection and use of denture base materials, considering their potential impact on biocompatibility and patient safety. We aimed to systematically examine the cytotoxic effects of thermoplastic denture base resins with the objective of consolidating and synthesizing the available evidence to enhance understanding and inform clinical practice.

## 2. Materials and Methods

The present review was performed according to the Preferred Reporting Items for Reviews and Meta-Analysis (PRISMA) 2020. Before initiation, a protocol including all aspects of the review methodology was made. Accordingly, a focused question was developed based on PICOS:

(P) Population: human gingival fibroblasts (hGF), human adipose tissue or human oral keratinocytes (IHOKs), human mesenchymal stem cells (hMSCs) isolated from patient tissue, or human amnion fibroblasts (HAFs) acquired from a pregnant woman or mouse fibroblasts (L929s).
(I) Intervention: thermoplastic or polyamide denture base resin specimens.
(C) Control: conventional polymethyl methacrylate (PMMA) denture base materials, heat-polymerized acrylic resin specimens, or untreated specimens.
(O) Outcome: cytotoxicity, cell viability, cell attachment, cell membrane damage.
(S) Study type: clinical, in vitro studies, in vivo studies.

The focused question developed is the following: Do thermoplastic denture base resins cause cytotoxicity or affect cell viability, attachment, or cell membrane damage?

### 2.1. Search Strategy

An electronic search was performed on Scopus, Web of Science, PubMed/Medline, and Google Scholar. Literature was searched for articles published up to September 2022 with no restrictions placed on the start date. Several search terms and search strategies were combined to identify studies. These include strategies to search the effects of thermoplastic resin materials on (1) cytotoxicity, (2) cell viability, (3) cell attachment, or (4) cell membrane damage. Forward citation tracking was conducted using Google Scholar. Full-text articles published in the English language were included. Case reports or case series were excluded if controls were not present. Case reports, systematic reviews, opinion articles, letters to the editor, and articles in languages other than English were excluded. 

### 2.2. Study Selection

Three authors (SP, GM, and GC) independently reviewed the search results for study selection. Duplicates and non-relevant articles were discarded. The researchers independently screened titles and abstracts of studies for eligibility and any disagreements were resolved through consensus with a fourth author (FL). The full text of relevant articles was examined for eligibility using the inclusion criteria. Manual Supplementary searches of the references of the selected articles were conducted for additional eligible studies. The search strategy is depicted in Table 1.

### 2.3. Data Extraction

The following characteristics were extracted by two authors (SP and GM) independently and verified by the third author (VB): author, year of publishing, country of origin, sample size, study design and methodology, intervention and outcome assessment, the outcome of the study, significant value, and inference of study. A customized template of extracted data was created manually to provide an overview of studies in a systematic manner.

### 2.4. Assessment of Study Quality

Based on the recommendations from the National Health and Medical Research Council for analyzing in vitro studies, the quality of the selected studies was assessed using relevant guidelines from the Toxicological Data Reliability Assessment Tool (ToxRTool) [31]. The tool assessed the inherent quality and reliability of the studies by examining five specific domains to assess whether eighteen criteria were met in the following areas: test substance identification, test system characterization, study design description, study result documentation, and plausibility of study design and data. Each criterion was scored as “yes” (1) or “no” (0). The absence of pertinent information in a selected study would result in a “no” judgment for the particular domain with a score of “0”. The combined scores were calculated and the reliability categorization of each study was carried out. Three ratings are possible based on the ToxRTool: reliable without restriction, reliable with restrictions, and not reliable. If any individual criterion was not met, a study could not be assigned as reliable irrespective of the numerical score. Overall inter-rater consistency was assessed using the kappa statistic.

### 2.5. Quality of Evidence for Outcomes in Summary of Findings Table

Relating to each outcome in the Summary of Findings, the quality of evidence was assessed using the evidence grading system GRADE, as described in Section 12.2 of the Cochrane Handbook for Systematic Reviews of Interventions [32]. The GRADE system was applied by one author (KA) and the quality of evidence for each outcome was then discussed with the other two authors (AB and GC). The final decision on ratings was made after discussion leading to a consensus. The certainty of the evidence was graded as high, moderate, low, and very low. Evidence for each outcome was graded as ‘high quality” at the start in the case of RCTs. The evidence rating was downgraded by one level for serious or two levels for very serious concerns regarding the study limitations, inconsistencies in the outcomes, indirectness of evidence, imprecision of effect estimates, or publication bias.

## 3. Results

**Figure 1:**
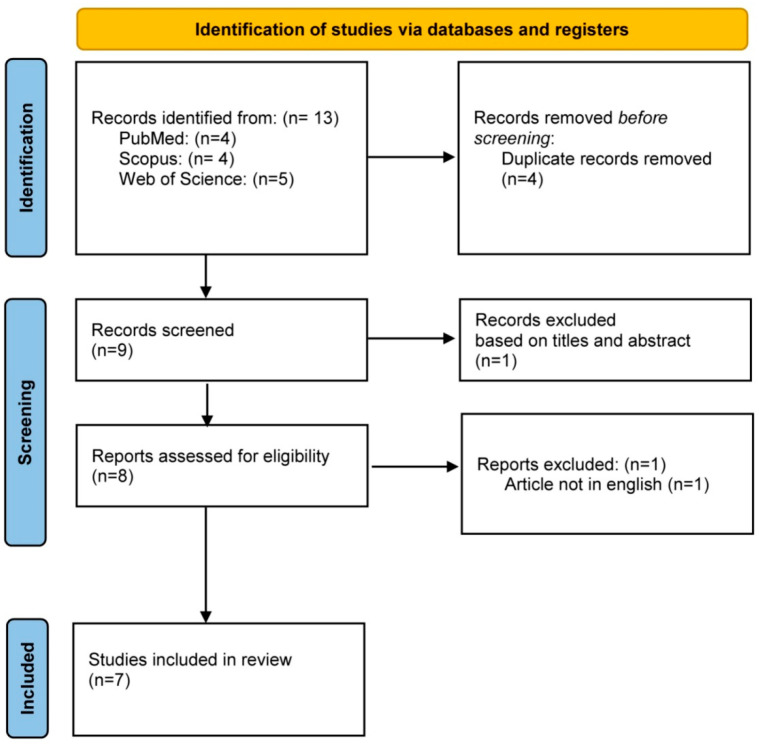
PRISMA flowchart.

The initial search results yielded 13 articles from the databases. After the removal of duplicates, the abstracts and titles were screened. Eight articles were assessed for eligibility by reviewing their full-text. One article was excluded as it was not published in English. In total, seven articles published between 2004 to 2022 were selected for inclusion in this review. The PRISMA flow diagram is shown in Figure 1.

Five of the seven studies included in the review were conducted in Asia (two in the Republic of Korea [23,33], two in Turkey [26,34], one in UAE [35], one study was conducted in the United States [36], and one in Africa (Egypt) [37].

### 3.1. Specimen Preparation

All seven studies included in the present review are in vitro studies that involved the use of cylindrical disc-shaped specimens of denture base resins for testing. Sample size for the studies ranged from 5 to 64 specimens across all seven studies.

### 3.2. Cell Lineage

Six of the seven included studies were conducted on human cell lines. One study cultured human amnion fibroblasts acquired from a pregnant woman for 14 days [27]. Three studies used gingival epithelial cells and fibroblasts cultured for 24 h and 48 h [36]; the fifth passage of human gingival fibroblasts [23]; and over 350 passages of oral gingival keratinocytes (IHOKs) immortalized by human papillomavirus, respectively [33]. One study cultured human mesenchymal stem cells at passage 8 [38] and one study used human adipose tissue [37]. Mouse fibroblast cell culture L-929 was used in two studies [33,34].

### 3.3. Medium Used

Only one study used BIOAMF-1 medium containing fetal calf serum [27], whereas the other six studies used Dulbecco’s Modified Eagle Medium with fetal bovine serum and antibiotics or antimitotics.

### 3.4. Comparator Used

All the studies compared the cytotoxicity of the thermoplastic denture resins with polymethyl methacrylate. Four studies also compared the cytotoxicity with different types of thermoplastic resins such as polyamide, nylon, thermoplastic acrylic, and polypropylene products [23,33,36]. Cengiz et al. compared the cytotoxicity with acrylic resin and Particulate filler resin composite [34].

### 3.5. Cytotoxicity Testing

Cytotoxicity and cell viability were assessed by reading the optical density of the resulting solution in six studies. One study used a Neural Red uptake assay, and the optical density of the resulting solution was read at 550 nm using a spectrophotometer [27]. One study used an EZ-Cytox Enhanced Cell Viability Assay Kit, and the optical density was measured on a spectrophotometer plate reader at 450 nm [23]. Two studies used MTT assay [34,36]; one study analyzed cell membrane damage measuring the release of cytoplasmic lactate dehydrogenase [36] and the other measured absorbance on a 570 nm absorbance-plate reader [34]. One study used WST-1 cytotoxicity assay and measured optical density on a spectrophotometer plate reader at 450 nm [38]. One study applied the MTS assay and optical absorbance was measured using a microplate reader at a wavelength of 490 nm. To confirm the cell viability tests, four studies performed live/dead analysis via confocal or UV microscope [33,36,37,38]. Cell attachment analysis was performed in one study [23].

### 3.6. Characteristics of Outcomes

All the studies included in the review showed that all denture base resins are cytotoxic to an extent. One study showed similar cytotoxic effect for all materials in short-term aging and highest toxicity levels were seen after 8 weeks of which heat-cured polymethyl methacrylate was most toxic [27]. One study showed that media conditioned with unpolished thermoformed nylon 6 polyamide denture material was significantly more toxic than the polished heat-polymerized polymethyl methacrylate and thermoformed nylon 12 composite polyamide conditioned media [36]. Two studies found no statistically significant differences between the cytotoxicity of thermoplastic acrylic resins, thermoplastic polyamide, and conventional heat-polymerized acrylic resins [23,38]. After 1 day, increased cell viability was seen in polyamides. After 6 days, decreased viability was seen in PMMA-based resin, whereas thermoplastic polyamide resins showed abundant cell attachment, thermoplastic polymethyl methacrylate resin showed similar but more stable pattern than heat-polymerized polymethyl methacrylate resin [23]. One study evaluated cell viability for 25, 12.5, and 6.25% extracts of tested samples after incubation at temperatures of 37 °C, 70 °C, and 121 °C. They found compromised IHOK viability in certain thermoplastic resins after incubation at 70 and 121 °C (32). Two studies found significant differences in the cell viability of human adipose tissue at 24 and 48 h and in mouse fibroblast cell culture L-929 for tested materials at incubation periods of 1, 24, 72 h, 1 week, and 2 weeks (*p* < 0.001) [34]. A summary of the characteristics of the included studies is shown in Table 2.

### 3.7. Quality Assessment

**Figure 2:**
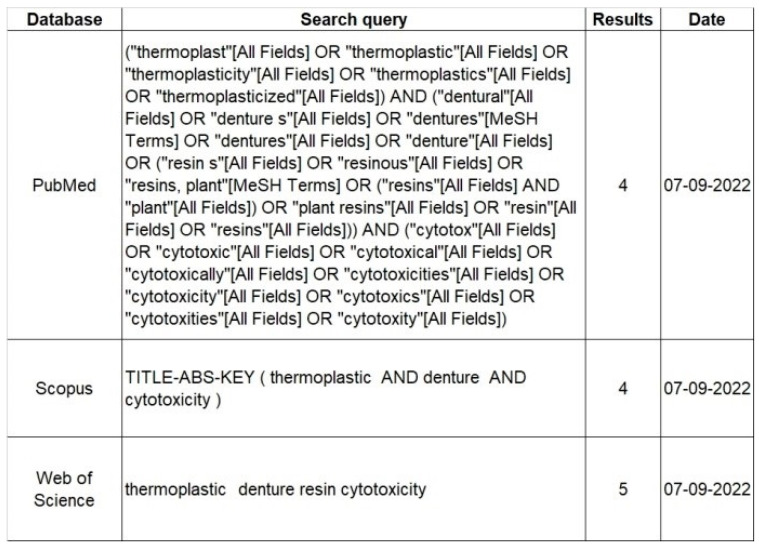
Search strategy.

The seven selected articles were submitted for assessment of the inherent quality of toxicological data, based on ToxRTool, employing an 18-point rating scale for in vitro studies. A majority of the studies included in this review showed a high risk of bias. Of the seven in vitro studies, two were determined to be ‘Reliable without restriction’, one was determined to be ‘reliable with restriction’, and four were determined to be ‘not reliable’, indicating a high risk of bias. In terms of the overall risk of bias, there were concerns regarding the test system, description of the study results, and reporting in the high-risk studies. Several examined studies suffered from methodological insufficiencies and a lack of transparent reporting. Several studies did not report on the number of replicate tests run and lacked a description of the study system. A detailed risk of bias based on the ToxRTool along with a summary assessment is presented in Figure 2 [39].

### 3.8. Certainty of Evidence

Our review examined seven studies with 461 samples. Based on GRADE, the overall quality of evidence in this study was low. This suggests limited confidence in estimating the cytotoxicity of denture base resin materials and raises doubts regarding the magnitude of the effect of the interventions examined. The reasons for downgrading the study were due to methodological insufficiencies, i.e., the studies were not randomized trials and the risk of bias. The majority of the involved studies either had some concerns or a high risk of bias. Table 2 shows the summary of findings.

## 4. Discussion

All the studies included in the current review conclude that all denture base resins, thermoplastic and conventional polymethyl methacrylate (PMMA), have toxic effects to a certain extent. Two studies showed that thermoplastic or polyamide resins have a comparable toxicity profile with PMMA denture base resins with no significant statistical difference [27,38], whereas one study showed that heat-cured acrylic resins are more cytotoxic than polyamide [37]. Another study showed that thermoplastic polyamide and PMMA-based thermoplastic acrylic resin resulted in better cell viability compared with PMMA-based conventional heat-polymerized acrylic resin; however, cell attachment was more abundant in polyamide than PMMA and more stable in thermoplastic rather than heat-polymerized acrylic resin [23]. Comparing the difference between polished and unpolished disks, it was found that thermoplastic nylon 6 polyamide, especially its unpolished disks, was more toxic than thermoplastic nylon 12 composite polyamide and heat-polymerized PMMA [36]. In the study comparing thermoplastic resin extracts from different incubation conditions, it was seen that polyamide resin-based products and thermoplastic acrylic-based products under different extraction temperatures showed lower cell viability than control and poly-propylene-based products [33]. However, at higher temperatures, 70 °C and 121 °C, resin-based thermoplastic resins were more cytotoxic than the control group [33]. It was also found that the representative conventional heat-polymerized PMMA resulted in around 100% cell viability in both human oral gingival keratinocytes and mouse fibroblasts L929s, which is in contrast with existing literature [40].

Evaluating biocompatibility is a complex process. Several test methods such as in vitro cell cultures and in vivo animal tests are used to evaluate the biocompatibility of material before application in patients. Though these tests elicit a biological response from materials, they cannot completely define the biocompatibility of the material. For testing the cytotoxicity of chemicals, in vitro cell viability and cytotoxicity assays with cultured cells are commonly used. These assays are rapid and inexpensive tests that exempt the use of animal models. These are based on various cell functions such as membrane permeability, enzyme activity, cell attachment, Adenosine triphosphate production, and nucleotide uptake. These assays screen compounds to test if they affect cell proliferation or show cytotoxic effects that ultimately cause cell death [41].

For colorimetric detection of viable cells, tetrazolium reagents such as MTT, MTS, XTT, and WST-1 have been used. MTT (3-(4,5-dimethylthiazol-2-yl)-2,5-diphenyltetrazolium bromide) is positively charged and penetrates viable eukaryotic cells readily, whereas MTS, XTT, and WST-1 (2-(4-iodophenyl)-3-(4-nitrophenyl)-5-(2,4-disulfophenyl)-2*H* tetrazolium monosodium salt) are negatively charged; hence, they do not penetrate cells as readily. The latter is used with an intermediate electron acceptor such as mPMS (1-methoxy-5-methyl-phenazinium methyl sulfate) that transfers electrons from the cytoplasm of the cell or its plasma membrane and reduces tetrazolium into a highly water-soluble colored formazan product [42,43]. MTT assay was the first homogenous assay developed for a 96-well format for high-throughput screening (HTS) [44]; it measures the activity of mitochondrial enzymes like that of succinate dehydrogenase to evaluate the mitochondrial function of cells and, hence, the cell viability [45]. The MTT assay reflects cell metabolism and not cell proliferation, whereas WST-1 is a cell proliferation assay [42,46]. This diverse range of techniques enabled a comprehensive evaluation of cytotoxic effects.

All but one study included in the review used human cells such as gingival fibroblasts (hGF), oral keratinocytes (IHOKs), mesenchymal stem cells (hMSCs), or amnion fibroblasts (HAFs) for evaluating cell viability. Mouse fibroblasts (L929s) were cultured in two studies [33]. Primary cell culture or immortalized cells from the target tissue provide tissue-specific sensitivity. The primary culture is obtained by culturing cells that are obtained from tissues or organs for more than 24 h, such as pulp fibroblasts. Isolating and culturing primary cultures from humans is difficult. Furthermore, being obtained from different individuals, their functional states are reflected differently [47]. Primary cell cultures are transformed into persistent cell lines that can proliferate indefinitely and have genetic and metabolic stability [48]. Hence, permanent cell lines are recommended for screening the toxicity of dental materials [49]. Human mesenchymal stem cells (hMSCs) are multipotent self-renewing progenitor cells that secrete growth factors. These cells can differentiate into various cell types such as chondrocytes, adipocytes, and osteoblasts that can be easily isolated and expanded [50]. As compared with other mammalian cells, human cells show different biological responses to toxic components [51,52]. The variations in cytotoxicity results could be due to the permeability of cell membranes, intracellular availability, and extracellular interactions with the components released from the materials [53]. Animal cell culture was used as a standard for cell viability, and a number of viable cells included Mouse fibroblast culture L-929 [54]. The majority of the included studies (six out of seven) utilized human cell lines, providing relevant data in the context of human cytotoxicity. Different cell types were employed, such as human amnion fibroblasts, gingival epithelial cells and fibroblasts, human mesenchymal stem cells, and oral gingival keratinocytes [55]. This diversity allows for a comprehensive evaluation of cytotoxicity across various cell lineages.

Extraction of specimens is a complex procedure that depends on factors such as temperature, time, surface area, volume, extraction vehicle, and equilibrium phase of the material. It is usually performed at 37 °C for 24 h in distilled water or a culture medium as the vehicle because, in the oral cavity, resins are exposed to body temperature (37 °C). The temperature can be increased to mimic clinical conditions. It was observed that when incubated at 37 °C, none of the tested thermoplastic denture resins showed severe toxicity (cell viability <70%), whereas some thermoplastic resins showed compromised cell viability when incubated at 70 and 121 °C [33]. This infers that some thermoplastic resins may elicit concerns regarding their safe applications in terms of the reaction of the oral mucosa to the chemicals released when resins are exposed to clinical conditions such as intake of hot food or beverages where the temperature is elevated.

When comparing the toxicity of polished and unpolished denture surfaces, there was a decrease in the cytotoxicity of polished denture resin-conditioned media compared with unpolished resin surface-conditioned media [36]. When thermoplastic resins were stored in artificial saliva for a long duration without polishing, they exhibited cell viability as low as 40% [27]. This difference could be associated with finishing and polishing procedures [56,57]. There is a possibility that certain toxic substances may be removed from the surface or become inactive [57].

The difference in toxicities of different denture materials at different times is related to the composition of denture base material, degree of polymerization, density of the material, toxic substances removed, rate of removal, and mechanism of toxicity.

Several studies show that within the first 48 h, the toxic effects of resins are maximum on the oral tissues. Hence, it is suggested to store the resins in water for at least 48 h before the delivery of the prosthesis for the removal of toxic substances. However, it has been reported that leaching of cytotoxic components may occur even after two weeks [58]. In the studies included in the present review, it was observed that after 24 h of aging, denture base resins and the control group showed similar toxic effects [23,27] and reached the highest toxicity levels after aging for 8 weeks [27]. After 24 h, thermoplastic polyamide resin showed the smoothest and most efficient cell attachment; after 6 days of incubation, thermoplastic polyamide resin showed the most abundant cell attachment followed by thermoplastic acrylic resin and heat-polymerized PMMA resin, respectively [23]. The late release of components affects long-term cytotoxicity. The correlation of this in vitro outcome to clinical oral conditions may be affected by pH, thermal changes, and occlusal forces. The chemical properties and surface characteristics may change over time as compared to the standard experimental setup where the temperature and moisture are constant and pH is stable [56]. The study by Lee et al. is the only study to evaluate the cytotoxicity of thermoplastic denture resin at high temperature [33].

To date, the cytotoxic substances released from thermoplastic denture resins have not been identified. Since thermoplastic resins polymerize by addition, the linear chains that are held by weak van der Waals forces can move freely without degrading at high temperatures. On the other hand, conventional acrylic resins polymerize by condensation and degrade at high temperatures. Hence, thermoplastic resins are more biocompatible because of their structure and addition polymerization [33,59]. However, detection and analysis of cytotoxic components released in the extracts of thermoplastic denture resins at high temperatures that can cause toxic reactions is necessary [59].

All the in vitro studies in the present review resulted in acceptable cytotoxicity of thermoplastic denture base resin, with some concern for high-temperature conditions. Studies have revealed a negligible influence on the viability of human gingival or amnion fibroblasts and mouse fibroblasts (L929s). Hence, these materials are considered non-cytotoxic.

### 4.1. Overall Completeness and Applicability

All of the studies included are in vitro studies using independent cell lines that cannot entirely simulate immune response, cytotoxic reactions, and hypersensitivity, which comprises multiple cell types and extracellular matrices [60]. In the included studies, the cultured cells in each study differed, the incubation period in each study differed, and the cell viability assays in each study used for outcome assessment are different.

Despite the overall acceptable cytotoxicity observed in vitro for thermoplastic denture base resins, with some concerns for high-temperature conditions, it is important to note that all the studies included in this review are in vitro studies using independent cell lines. These studies cannot fully replicate the complex immune responses, cytotoxic reactions, and hypersensitivity reactions that occur in vivo, which involve multiple cell types and extracellular matrix. The lack of in vivo or long-term clinical studies evaluating the cytotoxicity of thermoplastic denture base materials highlights the need for further research in this area.

### 4.2. Quality of Evidence

Methodological quality is referred to as “reliability” in toxicology [61]. We examined the standards and rigor for reporting the results of the research on the primary outcome of ‘cytotoxicity of denture base resins’. On the basis of the criteria used for the critical appraisal of the studies, only two studies were found to be reliable without restrictions, i.e., with a low risk of bias. We judged most studies to be at a high risk of bias due to their ‘reliable with restriction’ and ‘not reliable’ ratings. This was due to the volume of studies that suffered from methodological insufficiencies and a lack of transparent reporting. Several studies omitted information regarding the number of replicates run and positive controls used. This raises concerns about the way the trials were conducted and the reliability of the results reported by the investigators. These concerns are reflected in our judgement regarding the low certainty of the evidence for the cytotoxic effect of thermoplastic or polyamide denture base resin on cell viability, cell attachment, and cell membrane damage. The low quality of evidence is insufficient to enable robust conclusions to be drawn. Future studies must adhere to methodological standardizations, as recognized by the International Standard Organization, to evaluate the quality of products.

Every step of this review was carried out with the aim of minimizing bias. A comprehensive and sensitive search strategy with multiple independent authors served to identify studies for inclusion in this review. There was no restriction on the publication date. To avoid any selection bias, the authors independently evaluated the eligibility of articles to be included. However, one limitation in our review was that only English language studies were included. Thus, this review may not be exhaustively comprehensive due to the exclusion of articles published in other languages. Further high-quality trials using multiple assessment protocols are necessary before definitive universal guidelines can be issued.

## 5. Conclusions

This review evaluated the cytotoxic effects of thermoplasticized denture base resins on human gingival epithelium and fibroblasts, human amnion fibroblasts, and mouse fibroblasts. Based on the limited evidence available, thermoplasticized denture base resins are biocompatible and non-cytotoxic. Thermoplastic resins have similar or better toxicity profiles and cell attachment compared with conventional PMMA resins. When incubated at a higher temperature, thermoplastic resins show severe cytotoxicity. However, analysis of components released by these resins is important to evaluate the possible toxic reactions that can be caused by them. Further long-term in vivo and clinical assessments are necessary to corroborate laboratory findings with clinical outcomes.

## Figures and Tables

**Table 1:** Characteristics of selected studies.

Author, Year, and Country	Sample Size	Study Design	Cell Lines	Culture Medium	Incubation Period	Outcome Assessment	Outcome	Inference
IH Uzun et al., 2013. Turkey	*n* = 20 for each group and was divided into four sub-groups (*n* = 5)	G1: heat-cured Polymethyl methacrylate specimens processed using a conventional pressure-pack technique G2: self-cured Polymethyl methacrylate specimens polymerized at room temperature G3: polyamide resin Deflex specimens plasticized at 270C in injection flasks	Human amnion fibroblasts acquired from pregnant woman cultured for 14 days	BIOAMF-1 medium	24 h, 1 week, and 8 weeks	Neutral Red uptake assay and optical density of resulting solution measured at 550 nm using spectrophotometer.	Cell viability similar (*p* > 0.05) for all materials initially. After 24 h, Deflex more toxic than control group (*p* < 0.05). After 1 week, all materials reached highest values, not statistically different from initial and 24 h cell viabilities. After 8 weeks, all materials more toxic than control group, initial, 24 h, and 1-week aging times (*p* < 0.05). QC-20 was the most toxic material after the 8-week aging time and significantly different from Deflex and SC Cold Cure (*p* < 0.05).	Polyamide specimens had a comparable toxicity profile with the conventional Polymethyl methacrylate denture base materials in all tests. All materials showed similar toxic effects according to the control group in short-term aging periods. All tested materials reached the highest toxicity levels after the 8-week artificial aging time.
Wicks R et al., 2015. United States	*N* = 64 disks for each test denture material	G1: heat-polymerized polymethyl methacrylate (Lucitone 199, Dentsply) G2: thermoformed nylon 6 polyamide (ValplastTM, Valplast International) G3: thermoformed nylon 12 composite polyamide (DuraflexTM, Myerson)	Human gingival epithelial cells and fibroblasts from gingival explants of healthy individuals with noninflamed gingiva	Dulbecco’s Modified Eagle Medium, with 10% newborn calf serum and 100 mg/mL gentamicin	1 and 7 days	Cell toxicity assessed by MTT cell viability assay. Cell membrane damage by release of cytoplasmic LDH. Confirmation by live/dead staining and observation under UV microscope.	Unpolished Valplast conditioned media were toxic; media from polished Lucitone and Duraflex were less toxic. After 7 days of incubation: Valplast unpolished conditioned media—only 1 to 2% of the cells viable; polished disk conditioned media—significantly less (*p* < 0.05) toxicity. Data from lactate dehydrogenase (LDH) assay and live/dead mammalian cell viability assay in agreement with MTT viability assay.	Conditioned media from unpolished Valplast denture material appeared to be significantly more toxic to gingival fibroblasts and epithelial cells when compared with the polished Lucitone disk conditioned media as well as the media obtained from Duraflex.
Jang DE et al., 2015. Korea	*n* = 5 disc specimens for each group for cytotoxicity test	G1: Paladent 20, a PMMA-based conventional heat-polymerized acrylic resin G2: Bio Tone, a thermoplastic polyamide resin G3: Acrytone, a PMMA-based thermoplastic acrylic resin	Fifth passage human gingival fibroblasts cultured and seeded in a 12-well plate at a density of 1.8 × 10^4^ cells/well and cultured for 24 h in incubator	DMEM containing 10% FBS and 1× antibiotic/antimycotic	1, 6, and 10 days	Cell viability assay: EZ-Cytox Enhanced Cell Viability Assay Kit and optical density measured at a wavelength of 450 nm using a microplate reader. Cell attachment analysis: incubated cells fixed with 2.5% glutaraldehyde and observed with FE-SEM at ×1000 magnification.	After 1 day, cell viability was unimpaired in specimens and control group; increased viability in polyamides. PMMA-based resin showed decreased absorbance and cell viability lower than polyamides on 6th day. On day 1, Bio Tone showed smoothest pre-test surface and most efficient cell attachment; Acrytone and Paladent 20 showed moderate and poor cell attachment. On day 6, Bio Tone had richest hGF cell attachment. On day 10, BioTone showed most abundant cell attachment, Acrytone and Paladent 20 showed similar cell attachment, Acrytone showed more stable pattern.	Cytotoxicity of thermoplastic acrylic resins is similar to that of the thermoplastic polyamide and conventional heat-polymerized acrylic resins.
Al-Dharrab A and Shinawi LA, 2016. Saudi Arabia	*n* = 10 disc-shaped specimens for both groups	G1: heat-cured acrylic resin (vertex—Dental B.V, Zeist, Netherlands) G2: thermoplastic acrylic resin (Bre. flex polyamide, Bredent, Gmbh. Co.K.G. Senden, Germany) using the injection molding technique	Human mesenchymal stem cells at passage 8	Dulbecco’s Modified Eagle Medium with 15% fetal bovine serum and 1% antibiotic	24 h	WST-1 cytotoxicity assay and optical density measured on a spectrophotometer plate reader at 450 nm. Cell viability confirmed using live/dead fluorescent staining and photographed at 10× magnification.	Survival cell rate in both groups after 24 h was higher than control with more survival cell rate of hMSCs in G1 (no statistical significance). Green fluorescence cell observed in Groups 1 and 2 with fewer scattered red fluorescence cell in G2; difference was not significant in both groups.	Polymerization method used in both groups had no effect on the cytotoxicity or biocompatibility of denture base resins.
Lee JH et al., 2017. Korea	*n* = 6 disc specimens for each group	G1: polyamide resin-based products—Smile tone (ST), Valplast (VP), and Luciton FRS (LF) G2: thermoplastic acrylic resin-based products—Acrytone (AT) and Acryshot (AS) G3: polypropylene-based products—Unigum (UG) G4: conventional heat-polymerized acrylic resin-based products—Vertex (RS)	Oral gingival keratinocytes (IHOKs 55–60 passages) immortalized by human papillomavirus and confirmed to express epithelial markers over 350 passages; L929 mouse fibroblast cells (5–10 passages)	DMEM/F-12 3:1 mixture and RPMI 1640 containing 10% fetal bovine serum and 1% penicillin/streptomycin	24 h	Cell viability by MTS assay. Optical absorbance measured using microplate reader at a wavelength of 490 nm. Confirmation by live/dead analysis performed via confocal microscopy.	Cell viability more than 70% in all groups and extraction conditions. In 50% extract co-culture, cell viability of VP extracts at 70 °C and AT at 121 °C was significantly lower than control (0% extract, *p* < 0.05); 37 °C LF extract yielded 72.7 ± 4.3% cell viability. Under the 50% extract co-culture, LF extracts at 37 °C, VP at 70 °C, and AT at 121 °C—significantly lower cell viability than control (0% extract, *p* < 0.05). Cell viabilities measured for 25, 12.5, and 6.25% extracts of all tested samples were not significantly different from control, except 25% LF extract at 37 °C (0% extract, *p* > 0.05).	Severe cytotoxicity (less than 70%) was not detected in any tested thermoplastic denture base resins when IHOKs and L929s were subjected to extracts obtained after incubation at different temperatures (37 °C, 70 °C, and 121 °C). Compromised IHOK viability was detected in some thermoplastic resins following incubation at high temperatures (70 and 121 °C).
Elmwafy DA et al., 2019. Egypt	*n*= 30 disc-shaped specimens for each resin	Group I: 70 specimens constructed of heat-cured acrylic resin Group II: 70 specimens constructed of flexible resin Subgroup A: addition of silver vanadate nanorods Subgroup B: addition of titania nanorods Division 1: 0 wt% Division 2: 1 wt% Subdivision 1: 24 h Subdivision 2: 48 h	Cryotube of cell line from human adipose tissue	DMEM + 10% FBS medium	24 and 48 h	Cell toxicity: Tryptan-blue staining after 24 and 48 h of incubation of the discs with cells.	Highest mean value (82%) for heat-cured acrylic resin with 1% wt silver vanadate nanorods at 48 h; lowest mean value (0%) for thermoplastic resin with 0% wt titania nanorods at 24 h. Four-way ANOVA showed significant differences (*p* < 0.05) between all specimens and the interaction between them.	Cytotoxicity of heat-cured acrylic resin specimens was higher than polyamide. Silver nanorods have an adverse cytotoxic effect on both flexible and heat-cured acrylic resins. Titania nanorods are biocompatible materials and have no cytotoxic effect with flexible resin.
Cengiz S et al., 2022. Turkey	*n* = 10 disk-shaped specimens	Acrylic resin: Vertex (V), Orthocryl (O), Imident (I), Paladent (P), Meliodent (M) Particulate filler resin composite: Signum (S), Adoro (A), Tescera (T) Thermoplastic material: Bioplast (B) The specimens were divided into two groups to be stored either in artificial saliva (AS) and AS with melatonin (ASM)	Mouse fibroblast cell culture L-929	Dulbecco’s Modified Eagle Medium with 10% fetal bovine	1, 24, 72 h, 1 week, and 2 weeks	MTT cell viability assay and absorbance measured on 570 nm absorbance-plate reader	Significant difference for tested materials at each incubation period (*p* < 0.001). Interaction terms between the tested materials and the incubation periods were not significant (F = 0.864; *p* = 0.691). Three-factor interaction (group, material, and incubation time) was not significant (F = 1.221; *p* = 0.196). No significant difference between the O, V, I materials in ASM at 1 h incubation period, the absorbent index values increased for M, A, T, B, S, and *p* materials.	At 1 h, all auto-polymerized acrylic resin specimens (M) showed no change in cytotoxicity level. Heat-polymerized, particulate filler composite resins and the thermoplastic materials presented decreased cytotoxicity at 1 h.

**Table 2:** Summary of findings table.

Quality Assessment						Summary of Findings		
Outcome	Risk of Bias	Inconsistency	Indirectness	Imprecision	Publication Bias	Impact	No. of Studies	Certainty of Evidence (GRADE)
Cytotoxic effect of thermoplastic or polyamide denture base resin on cell viability, cell attachment, cell membrane damage	Serious	Not serious	Not serious	Not serious	Not serious	Our confidence in the effect estimate is limited	7	Low
